# Mutual Inhibition of Antithrombin III and SARS-CoV-2 Cellular Attachment to Syndecans: Implications for COVID-19 Treatment and Vaccination

**DOI:** 10.3390/ijms25147534

**Published:** 2024-07-09

**Authors:** Anett Hudák, Dávid Pusztai, Annamária Letoha, Tamás Letoha

**Affiliations:** 1 Pharmacoidea Ltd., 6726 Szeged, Hungary; anett.hudak@pharmacoidea.eu (A.H.); david.pusztai@pharmacoidea.eu (D.P.); 2 Department of Medicine, Albert Szent-Györgyi Clinical Center, Faculty of Medicine, University of Szeged, 6720 Szeged, Hungary; letoha.annamaria@med.u-szeged.hu

**Keywords:** antithrombin III, heparan sulfate proteoglycans, syndecans, SARS-CoV-2, spike protein cellular entry

## Abstract

Antithrombin III (ATIII) is a potent endogenous anticoagulant that binds to heparan sulfate proteoglycans (HSPGs) on endothelial cells’ surfaces. Among these HSPGs, syndecans (SDCs) are crucial as transmembrane receptors bridging extracellular ligands with intracellular signaling pathways. Specifically, syndecan-4 (SDC4) has been identified as a key receptor on endothelial cells for transmitting the signaling effects of ATIII. Meanwhile, SDCs have been implicated in facilitating the cellular internalization of SARS-CoV-2. Given the complex interactions between ATIII and SDC4, our study analyzed the impact of ATIII on the virus entry into host cells. While ATIII binds to all SDC isoforms, it shows the strongest affinity for SDC4. SDCs’ heparan sulfate chains primarily influence ATIII’s SDC attachment, although other parts might also play a role in ATIII’s dominant affinity toward SDC4. ATIII significantly reduces SARS-CoV-2′s cellular entry into cell lines expressing SDCs, suggesting a competitive inhibition mechanism at the SDC binding sites, particularly SDC4. Conversely, the virus or its spike protein decreases the availability of SDCs on the cell surface, reducing ATIII’s cellular attachment and hence contributing to a procoagulant environment characteristic of COVID-19.

## 1. Introduction

Antithrombin III (ATIII) is a crucial inhibitor from the serpin superfamily that regulates the activity of serine proteases in the blood coagulation system [1]. Synthesized in the liver, ATIII is renowned for its potent anticoagulant properties. It interacts with the coagulation system’s activated proteases and cell-surface heparan sulfate proteoglycans (HSPGs) [2]. Notably, the D-helix of ATIII binds to both therapeutic heparins and HSPGs on vascular endothelial cells, enhancing ATIII’s ability to inhibit coagulation proteases [1]. ATIII’s attachment to HSPGs also elicits a robust anti-inflammatory response in vascular endothelial cells involving the synthesis of prostacyclin (PGI2), which inhibits nuclear factor-κB (NF-κB) and the production of proinflammatory cytokines and adhesion molecules [3].

Syndecans (SDCs) are the only transmembrane HSPGs in nearly all nucleated cells of vertebrates and invertebrates [4]. SDCs are pivotal in transmitting extracellular signals into the cell interior and modulating cell adhesion, migration, junction formation, polarity, and differentiation [5]. The universally expressed isoform syndecan-4 (SDC4) has been identified as a specific ATIII receptor on endothelial cells that transmits the signaling effects of ATIII to relevant second messenger molecules within the cell’s signal transduction pathways [3,4]. This interaction is vital for modulating the inflammatory responses of endothelial cells and leukocytes, which ATIII binds to through SDC4.

Beyond its anticoagulant and anti-inflammatory roles, ATIII shows promise in defending against infectious agents and exhibits significant antimicrobial properties [2]. ATIII levels are often reduced in systemic inflammatory diseases due to its consumption by the coagulation system and various immunological processes. This decrease in ATIII can compromise the body’s ability to manage inflammation and infection effectively [2,6]. Furthermore, ATIII binds to glycocalyx structures such as SDC4, enhancing its ability to modulate the inflammatory responses of endothelial cells and leukocytes. This interaction is vital for maintaining endothelial integrity during inflammatory states induced by viral infections [7,8]. Studies on patients with COVID-19 further underscore the clinical relevance of ATIII in viral infections. Lower levels of ATIII have been significantly associated with higher mortality rates in critically ill COVID-19 patients, even those receiving therapeutic doses of low molecular weight heparin [9,10]. This association highlights ATIII’s potential as a marker for inflammation and infection and as a predictor of survival outcomes in patients with acute liver failure and severe viral pneumonia [11,12,13,14]. These findings emphasize the need for further research to explore ATIII’s full therapeutic potential, particularly its role in managing hypercoagulability and inflammatory responses in severe viral infections.

Cell surface HSPGs, on the other hand, play a pivotal role in the cellular entry of various pathogens, including SARS-CoV-2 (SCV2) [15]. Numerous studies have underscored the crucial function of heparan sulfate (HS) in SCV2 infection [16,17,18].

Previously, we explored the involvement of SDCs in SCV2’s cellular entry [19]. Our studies showed that SCV2 enters cells after attaching to SDCs through the spike protein’s S1 subunit. Among the SDCs, SDC4 has been identified as the most efficient in mediating this uptake. This interaction facilitates the virus’s entry into cells, highlighting the critical role of SDCs, especially SDC4, in the SCV2 infection process.

Considering ATIII’s affinity toward SDCs, we explored ATIII’s influence on SCV2’s cellular internalization. Utilizing our previously established SDC-overexpressing cell lines, we investigated ATIII’s interaction with SDCs and its effect on SCV2’s cellular uptake. Moreover, we observed a cellular phenomenon that might account for the thrombogenic activity of SCV2 and its spike protein. Although further in vivo research is eminent, our findings deepen the understanding of the molecular interplay of ATIII, HSPGs, and SCV2, hence facilitating the development of novel COVID-19 treatment strategies and safer COVID-19 immunization protocols.

## 2. Results

### 2.1. Effect of SDC Overexpression on ATIII’s Cellular Attachment

**Figure 1:**
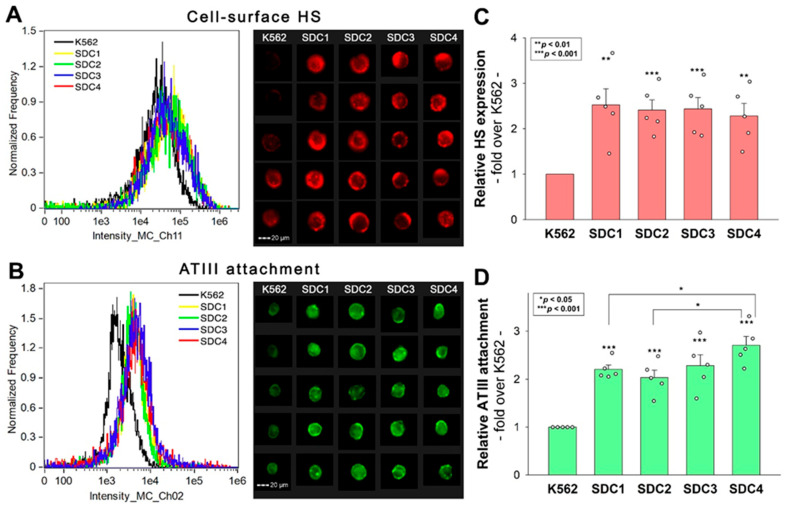
ATIII attachment of SDC transfectants. WT K562 cells and stable SDC transfectants were incubated with 5 U/mL of recombinant ATIII for 30 min at 37 °C. ATIII’s cellular attachment was then analyzed with imaging flow cytometry using an AF488-labeled ATIII antibody. (**A**,**B**) Representative flow cytometry histograms and cellular images showing the HS expression (**A**) and ATIII attachment (**B**) of WT K562 cells and SDC transfectants as measured with imaging flow cytometry using fluorescent HS and ATIII antibodies. Scale bar = 20 μm. (**C**,**D**) Detected fluorescence intensities normalized to HS- or ATIII antibody-treated WT K562 cells as standards. The bars represent the mean + SEM of five independent experiments (data are represented as dots). Statistical significance was assessed using ANOVA. * *p* < 0.05; ** *p* < 0.01; *** *p* < 0.001.

SDCs serve as ATIII’s key HSPG attachment sites on the cell surfaces [20]. However, comparative analyses on ATIII’s affinity toward SDC isoforms are still lacking. Thus, we analyzed ATIII’s attachment to the various SDC isoforms by utilizing stable SDC transfectants, specifically overexpressing each SDC isoform with even HS levels (Figure 1A,C) [21,22]. The even HS expression of the utilized SDC transfectants enables the analysis of ATIII’s interaction with SDCs beyond HS chains. Furthermore, these SDC transfectants were developed in K562 cells, which is a human cell line characterized by a lack of HSPGs except for minor amounts of endogenous betaglycan [23,24]. Due to their minimal HSPG content, using wild-type (WT) K562 cells as controls can be considered equivalent to employing SDC knockout (KO) cell lines. Thus, stable SDC transfectants and WT K562 cells (serving as controls) were incubated with ATIII at a concentration of 5 U/mL at 37 °C for 30 min. ATIII’s cellular attachment was then detected by incubating the cells with an ATIII-specific Alexa Fluor 488 (AF488)-labeled antibody and measuring the ATIII-specific fluorescence with imaging flow cytometry. Imaging flow cytometry showed that SDC overexpression increased ATIII attachment (Figure 1B,D). Among the SDC transfectants, SDC4 showed the highest ATIII-dependent fluorescence, indicating the most robust ATIII binding. (ATIII did not exhibit any cytotoxic effects [Figure S1]).

### 2.2. Removal of HS with Heparinase Reduces ATIII’s Cellular Attachment 

**Figure 2:**
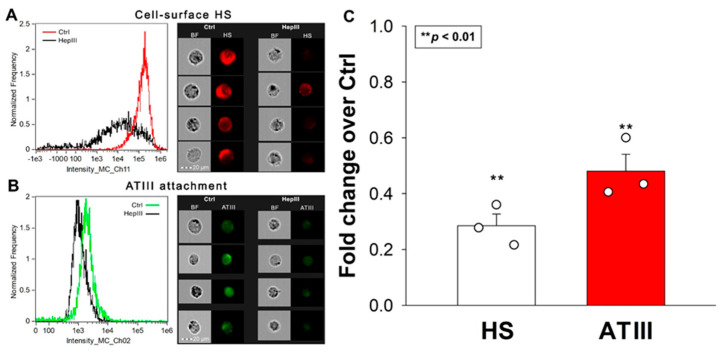
Removal of HS with HepIII reduces ATIII’s cellular attachment. SDC4 transfectants were incubated with or without HepIII for 4 h at 37 °C, followed by 30 min of ATIII treatment (at a concentration of 5 U/mL). ATIII attachment was then analyzed with imaging flow cytometry using primary and fluorescently labeled secondary antibodies. (**A**,**B**) Representative flow cytometry histograms, brightfield (BF) and fluorescent cellular images showing cell-surface HS content and ATIII attachment of SDC4 transfectants preincubated with or without HepIII. Scale bar = 20 μm. (**C**) Detected fluorescence intensities normalized to HepIII-untreated SDC4 transfectants as standards. The bars represent the mean + SEM of three independent experiments (data are represented as dots). Statistical significance vs. standards was assessed with ANOVA. ** *p* < 0.01.

Experiments with SDC transfectants revealed that SDC4 exhibits the greatest affinity for ATIII. However, all SDC transfectants possessing a significantly higher cell-surface HS content than WT K562 cells showed increased ATIII binding compared to WT K562 cells. To examine the importance of HS in the interaction with ATIII, we next examined ATIII’s attachment to SDC4 transfectants after the enzymatic removal of cell surface HS using heparinase III (HepIII). Removing approximately 70% of HS with HepIII resulted in about a 50% reduction in ATIII attachment, highlighting the critical role of HS chains in mediating SDC4′s interaction with ATIII (Figure 2A–C). (As shown in Figure S2, HepIII incubation did not affect cellular viability).

### 2.3. ATIII Inhibits SCV2’s Cellular Entry into K652 Cells and SDC4 Transfectants

**Figure 3:**
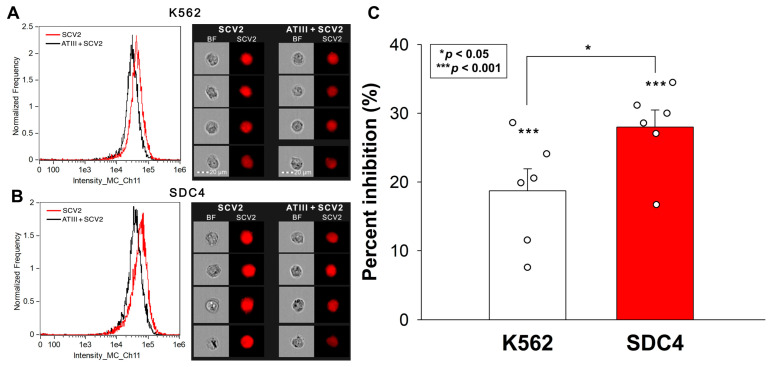
ATIII inhibits the WT SCV2’s cellular entry into K652 cells and SDC4 transfectants. WT K562 cells and stable SDC4 transfectants were exposed to 1 MOI of the heat-inactivated WT SCV2 with or without 5 U/mL of ATIII for 4 h at 37 °C. SCV2’s cellular uptake was then analyzed with imaging flow cytometry using primary spike and AF643-labeled secondary antibodies. (**A**,**B**) Representative flow cytometry histograms, BF and fluorescent cellular images showing the intracellular fluorescence of WT K562 cells and SDC transfectants treated with the inactivated virus. Scale bar = 20 μm. (**C**) Detected fluorescence intensities normalized to SCV2-treated but ATIII-untreated cells as standards. The bars represent the mean + SEM of six independent experiments (data are represented as dots). Statistical significance was assessed with ANOVA. * *p* < 0.05; *** *p* < 0.001.

We previously demonstrated that SDCs, particularly SDC4, facilitate SCV2’s cellular entry. Having identified SDC4 as the major receptor for ATIII among SDCs, we further investigated ATIII’s potential to inhibit the WT SCV2’s internalization into WT K562 cells and SDC4 transfectants. Our experiments confirmed the enhanced internalization of inactivated SARS-CoV-2 in SDC4 transfectants (Figure 3A,B). The co-incubation of SCV2 with ATIII resulted in a notable decrease in the virus’s cellular uptake. ATIII’s inhibitory effect was significantly more pronounced in SDC4 transfectants, reducing viral attachment and entry by approximately 27% compared to about 18% in WT K562 cells (Figure 3C).

It is worth noting that SCV2 did not affect the cellular viability of K562 cells and SDC4 transfectants at 1 MOI (Figure S3).

### 2.4. ATIII Inhibits SCV2’s Cellular Entry into Calu-3 Cells

**Figure 4:**
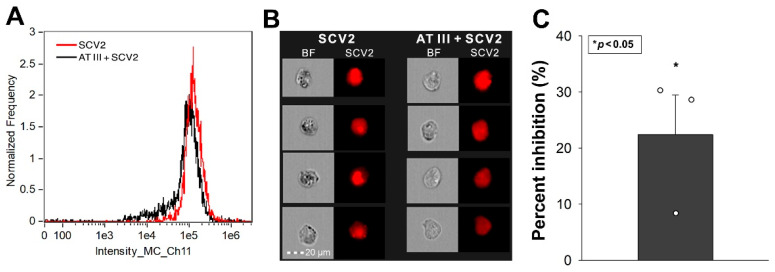
ATIII inhibits SCV2’s cellular entry into Calu-3 cells. WT Calu-3 cells were exposed to 1 MOI of the heat-inactivated WT SCV2 with or without 5 U/mL of ATIII for 4 h at 37 °C. Cellular uptake was then analyzed with imaging flow cytometry using primary and fluorescently labeled secondary antibodies. (**A**,**B**) Representative flow cytometry histograms, BF and fluorescent cellular images showing the intracellular fluorescence of cells treated with the virus. Scale bar = 20 μm. (**C**) Detected fluorescence intensities normalized to SCV2-treated but ATIII-untreated cells as standards. The bars represent the mean + SEM of three independent experiments (data are represented as dots). Statistical significance vs. standards was assessed with ANOVA. * *p* < 0.05.

Calu-3 cells, a cell line frequently used as a respiratory in vitro model, express high levels of SDC4 [25]. The Human Protein Atlas indicates that Calu-3 cells also express significant amounts of SDC1, low SDC2 and moderate SDC3 levels [26,27]. Given ATIII’s affinity for SDCs, we investigated its effect on SCV2’s cellular entry in Calu-3 cells. As illustrated in Figure 4A–C, ATIII significantly inhibited SCV2’s cellular entry into the cells. (SCV2 did not affect cellular viability [Figure S4]). 

### 2.5. SCV2 and the Spike Protein Are Internalized by HMEC-1 Cells

**Figure 5:**
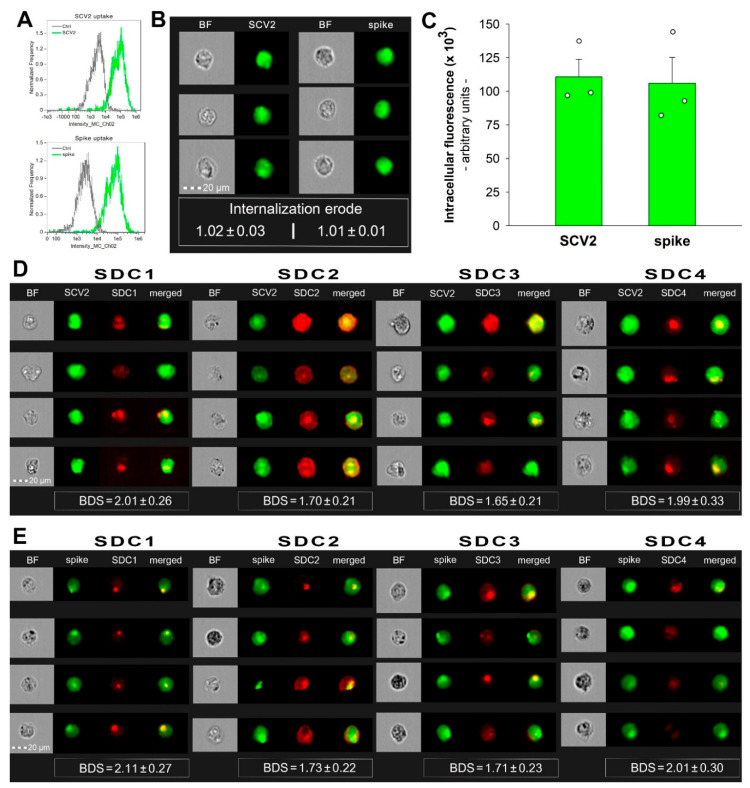
SCV2 and the spike are internalized by HMEC-1 cells and colocalize with SDCs. WT HMEC-1 cells were incubated with or without 1 MOI of the heat-inactivated WT SCV2 or recombinant WT spike protein for 6 h at 37 °C. After incubation, the cells were washed, trypsinized, fixed, permeabilized, and treated with antibodies specific for the spike glycoprotein (along with secondary AF 488-labeled antibodies). Some cells were also treated with APC-labeled SDC antibodies for colocalization analyses. Cellular uptake of SCV2 or the spike protein was then analyzed with imaging flow cytometry. (**A**) Representative flow cytometry histograms showing the intracellular fluorescence of HMEC-1 cells treated with or without WT SCV2 or spike. (**B**) BF and fluorescent cellular images of WT SCV2- or spike-treated HMEC-1 cells. Scale bar = 20 μm. The indicated internalization erode values represent the mean ± SEM of three independent experiments. (**C**) Detected fluorescence intensities of cells treated with either SCV2 or spike. The bars represent the mean + SEM of three independent experiments (data are represented as dots). (**D**,**E**) Colocalization of SDCs and WT SCV2 (**D**) or spike (**E**) in HMEC-1 cells. The indicated BDS values represent the mean ± SEM of three independent experiments.

Endothelial cells lining the blood vessels play a crucial role in ATIII’s anticoagulant function [28]. Endothelial cells’ HSPGs provide binding sites for ATIII and accelerate its ability to inactivate thrombin and other clotting factors [1]. HMEC-1 is an endothelial-like cell line with reported expression of SDC1, 2, and 4 (as shown in Figure 5, our imaging flow cytometry analysis revealed a low surface expression of SDC1 and SDC3 and high expression of SDC2 and SDC4 in these cells) [29]. According to our studies, HMEC-1 cells also readily internalize SCV2 or its spike protein (Figure 5A–E). Briefly, HMEC-1 cells were incubated with or without 1 MOI heat-inactivated WT SCV2 or recombinant WT spike protein (5 µM) for 6 h at 37 °C. After incubation, the cells were washed, trypsinized, fixed, permeabilized, and treated with spike glycoprotein-specific antibodies (along with secondary AF488-labeled antibodies). WT SCV2- or spike-treated cells exhibited markedly increased fluorescence with an internalization erode value of ~1, indicating an effective cellular entry ([30,31,32]). During imaging flow cytometry, the Bright Detail Similarity (BDS) feature of the Amnis IDEAS software was used to measure colocalization between SCV2 or its spike and SDCs (a BDS score of 2 or greater represents a high degree of overlap [19]). In the case of SDC1 and SDC4, the BDS scores with either SCV2 and spike were around 2, highlighting that the virus or its spike colocalizes with SDC1 or SDC4 during cellular uptake, suggesting a shared internalization mechanism (Figure 5D,E).

### 2.6. SCV2 or the Spike Protein Reduces SDCs on the Cell Surface

**Figure 6:**
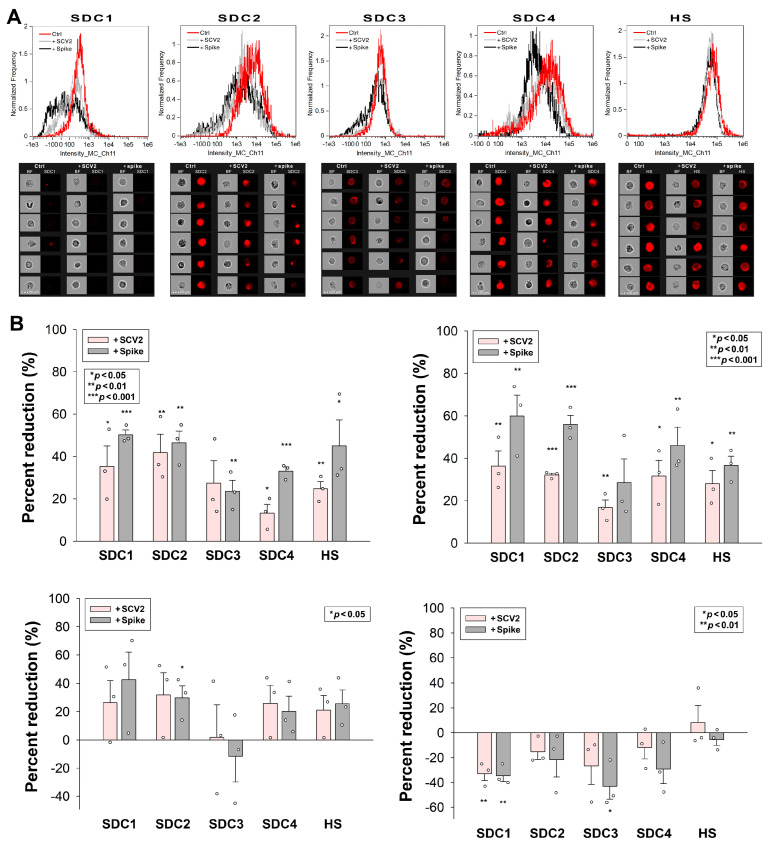
SCV2 or the spike protein reduces SDCs on the cell surface. HMEC-1 cells were incubated with or without heat-inactivated WT SCV2 or recombinant WT spike for 6, 24, 48, or 72 h at 37 °C. After incubation, the cells were washed and fixed, and surface SDC and HS expression were assessed with imaging flow cytometry, using fluorescently labeled SDC and HS antibodies. (**A**,**B**) Representative flow cytometry histograms (**A**), BF and fluorescent cellular images (**B**) showing SDC and HS expression of HMEC-1 cells incubated with or without heat-inactivated WT SCV2 or recombinant spike for 6 h. Scale bar = 20 μm. The effect of SCV2 or spike on surface SDC expression expressed as percent reduction. The bars represent the mean + SEM of three independent experiments (data are represented as dots). Statistical significance vs. untreated controls was assessed using ANOVA. * *p* < 0.05; ** *p* < 0.01; *** *p* < 0.001.

SDC ligands induce the accumulation and relocation of SDCs into lipid rafts, which is followed by the lipid raft-dependent internalization of the ligand–SDC complex [33]. Our study examined how the SDC-dependent entry of WT SCV2 or its spike protein influences the surface availability of SDCs in cells. HMEC-1 cells were incubated with or without heat-inactivated WT SCV2 (1 MOI) or the recombinant WT spike protein (at a concentration of 5 µM) for 6, 24, 48, or 72 h at 37 °C. After incubation, the surface SDC expression was assessed using imaging flow cytometry with fluorescently labeled SDC antibodies. Incubating HMEC-1 cells with WT SCV2 or its spike protein for 6 and 24 h significantly reduced the availability of various SDC isoforms on the cell surface (Figure 6A,B). However, after 48 h of incubation, SDC levels began to normalize. Interestingly, by 72 h, the number of surface-expressed SDC1 and SDC3 had increased (Figure 6B). (As shown in Figure S5, SCV2 or the spike did not affect cellular viability at the applied concentration, suggesting that SCV2’s or the spike’s effect on SDC levels does not stem from cytotoxicity).

### 2.7. SCV2 or the Spike Protein Interferes with ATIII’s Cellular Attachment

**Figure 7:**
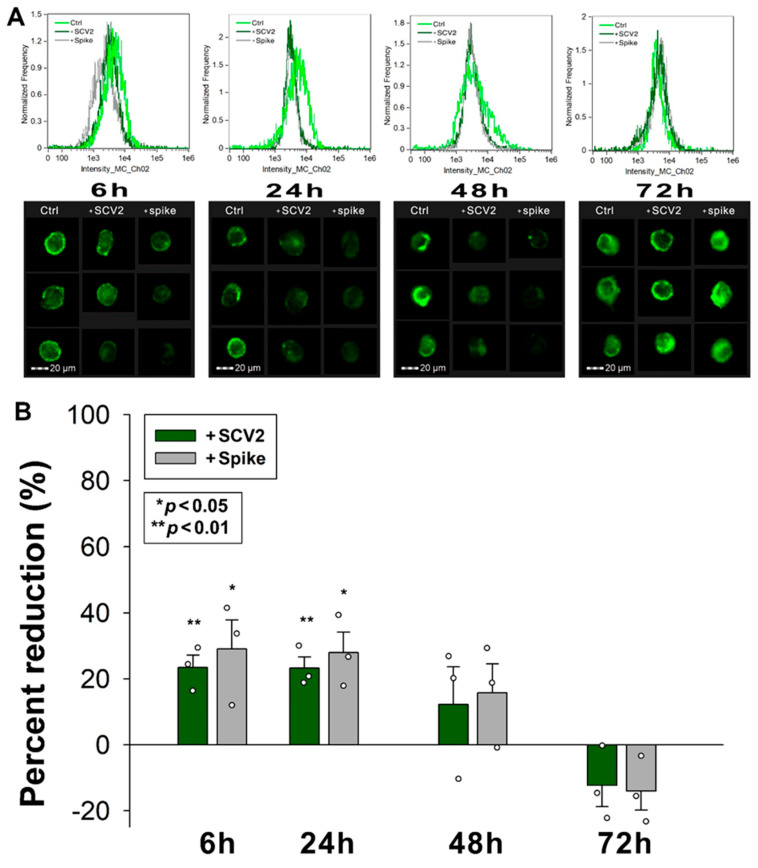
SCV2 or the spike protein interferes with ATIII’s cellular attachment. HMEC-1 cells were incubated with or without heat-inactivated WT SCV2 or recombinant WT spike for 6, 24, 48 or 72 h, in the presence of ATIII, at 37 °C. After incubation, the cells were washed, and ATIII attachment was assessed with imaging flow cytometry, using fluorescently labeled SDC and HS antibodies. (**A**,**B**) Representative flow cytometry histograms (**A**) and cellular images (**B**) showing ATIII attachment of HMEC-1 cells incubated with or without heat-inactivated WT SCV2 or recombinant spike. Scale bar = 20 μm. The effect of WT SCV2 or spike on surface SDC expression expressed as percent reduction. The bars represent the mean + SEM of three independent experiments (data are represented as dots). Statistical significance vs. untreated controls was assessed using ANOVA. * *p* < 0.05; ** *p* < 0.01.

We also explored how WT SCV2 or the spike protein influence ATIII attachment to HMEC-1 cells. The decrease in the availability of SDCs due to WT SCV2 and spike treatment also affected ATIII binding. Specifically, 6 and 24 h of SCV2 or spike incubation reduced ATIII attachment to HMEC-1 cells. However, after 48 h of incubation with SCV2 or spike protein, ATIII surface attachment began normalizing (Figure 7A,B).

## 3. Discussion

ATIII, a potent endogenous anticoagulant, has garnered significant attention in the context of COVID-19. Critically ill COVID-19 patients often exhibit decreased ATIII levels, stemming from the disease’s hypercoagulable and inflammatory state [9,34]. This condition leads to ineffective heparin treatment and elevated mortality rates, underscoring the importance of understanding ATIII’s role and potential impacts on SCV2 infection and COVID-19 prevention [35,36].

Both ATIII and SCV2 have reported affinities toward HSPGs, particularly SDCs [1,16,20,37,38,39]. Our study demonstrated that although ATIII is attached to all SDC isoforms, it exhibited the highest affinity toward SDC4. Given the consistent HS levels and indistinguishability of HS chains of HSPGs expressed in a given cell line, ATIII’s highest affinity toward SDC4 likely arises from interactions with HS-independent factors. Removing HS from SDC4 with HepIII markedly reduced ATIII attachment, highlighting the profound influence of HS chains in the SDC4–ATIII interaction.

ATIII also effectively decreased SCV2’s cellular entry, particularly in SDC4 overexpressing transfectants, underscoring ATIII’s affinity toward SDC4. Additionally, ATIII inhibited SCV2’s entry into WT Calu-3 cells, which is a cell line expressing high amounts of SDC1 and SDC4. In HMEC-1 endothelial cells expressing several SDC isoforms, SCV2 or its spike protein decreased the availability of SDC isoforms on the cell surface, reducing ATIII attachment at 6 and 24 h of incubation. Decreased ATIII attachment to endothelial cells can have significant in vivo effects related to the regulation of coagulation and inflammation [40]. As a crucial component of the anticoagulation pathway, decreased ATIII attachment leads to reduced inhibition of thrombin and Factor Xa, increasing the risk of thrombus formation. Moreover, ATIII’s anti-inflammatory and anti-apoptotic effects help preserve endothelial integrity [41,42]. Therefore, decreased endothelial ATIII attachment can compromise these protective effects, leading to endothelial dysfunction. With less ATIII available to protect the endothelium, vascular permeability may increase, contributing to edema and inflammation [43,44]. A heightened inflammatory response and a procoagulant environment can exacerbate conditions such as sepsis, where inflammation and coagulation are dysregulated. Endothelial cells with less bound ATIII may exhibit an increased expression of adhesion molecules, promoting the adhesion and migration of leukocytes into tissues, further driving inflammation [45]. Thus, by interfering with ATIII’s attachment to endothelial cells, SCV2 or its spike protein can disrupt the delicate balance between coagulation and anticoagulation as well as between proinflammatory and anti-inflammatory states. This disruption can increase the risk of thrombotic events, endothelial dysfunction, and inflammatory complications, contributing to various pathological consequences of SCV2 infection [46,47,48]. Moreover, the decreased endothelial attachment of ATIII can also explain how spike-encoding, genetic COVID-19 vaccines might increase thrombosis risk [49,50]. Free-floating spike was detected in individuals vaccinated with spike-encoding genetic (i.e., mRNA or adenovirus-based) COVID-19 vaccines [50,51,52,53,54]. Spike proteins entering the bloodstream can interfere with the binding of ATIII to endothelial cells, creating a procoagulant environment that favors coagulation.

Overall, these findings have significant practical implications. Understanding the role of ATIII in SCV2 infection could lead to new therapeutic strategies that enhance ATIII activity or prevent its displacement from endothelial cells, thereby reducing thrombotic and inflammatory complications in COVID-19 patients. Additionally, this knowledge could inform the design and administration of COVID-19 vaccines to minimize potential thrombotic risks, ensuring safer immunization strategies. Further research into the interactions between ATIII, HSPGs, and SCV2 could pave the way for novel treatments that mitigate the severe coagulopathic and inflammatory manifestations of COVID-19.

## 4. Materials and Methods

### 4.1. SDC Constructs

SDC transfectants, established in K562 cells (ATCC, Manassas, VA, USA; cat. no. CCL-243) were created as described previously [28,29].

### 4.2. Flow Cytometry Analysis of HS and SDC Expression

HS and SDC expression of SDC transfectants and HMEC-1 cells were measured with flow cytometry by using anti-human HS antibody (10E4 epitope; Amsbio, Abingdon, UK; cat. no. 370255-1) with AF647-labeled secondary anti-mouse IgM and respective isotype control (Thermo Fisher Scientific, Waltham, MA, USA, cat. no. 02-6800) and APC-labeled SDC antibodies as described previously [55]. SDC transfectants (created in K562 cells) with almost equal amounts of HS expression were selected for further uptake studies [19,21,22]. 

To examine the importance of HS in the interaction with ATIII, SDC4 transfectants were incubated with 1 unit/mL of HepIII (Sigma Aldrich, St. Louis, MI, USA; cat. no. H8891) for 4 h at 37 °C [56,57]. Surface HS expression was then assessed with imaging flow cytometry as described above.

In the case of SCV2- or spike-treated HMEC-1 cells, the cells were treated with or without 1 MOI of heat-inactivated SCV2 (strain: 2019-nCoV/USA-WA1/2020; ATCC, Manassas, VA, USA; cat. no. ATCC VR-1986HK) or N-terminally His-tagged recombinant SCV2 spike protein (Sino Biological, Beijing, China, cat. no. 40589-V08B1-100) at a concentration of 5 µM for 6, 24, 48, or 72 h at 37 °C. Surface SDC expression was then assessed as described above. 

For all SDC analyses, the relevant isotype controls were applied as described previously [55].

### 4.3. Flow Cytometry Analysis of ATIII Attachment

WT K562 cells, its SDC transfectants, and WT HMEC-1 cells were utilized to quantify ATIII attachment. Briefly, 3 × 10^5^ cells/mL in DMEM medium were exposed to ATIII at a concentration of 5 U/mL for 30 min at 37 °C. The cells were then fixed and treated with an AF488-labeled mouse monoclonal antibody raised against amino acids 331–400 mapping near the C-terminus of ATIII of human origin (Santa Cruz Biotechnology, Inc.; Dallas, TX, USA). After 1 h of incubation at room temperature, the cells were rinsed thrice with PBS containing 1% BSA and progressed toward flow cytometry. ATIII attachment was then measured with flow cytometry using an Amnis FlowSight imaging flow cytometer (Amnis Corporation, Seattle, WA, USA). A minimum of 5000 events per sample were analyzed. Appropriate gating in a forward-scatter-against-side-scatter plot was utilized to exclude cellular debris and aggregates. Fluorescence analysis was conducted using the Amnis IDEAS^®^ analysis software (version 6.2.187.0, Amnis Corporation, Seattle, WA, USA).

### 4.4. Flow Cytometry Analysis of Virus and Spike Uptake

WT K562 cells, its SDC4 transfectants, WT Calu-3 and HMEC-1 cells were utilized to quantify SCV2 or spike internalization. Briefly, 3 × 10^5^ cells/mL in DMEM medium were exposed to either 1 MOI of SCV2 or the spike protein (5 µM) for 4 or 6 h at 37 °C. After incubation, the cells were washed and trypsinized (with the method described by Nakase et al. [28,40,41]) to remove the extracellularly attached viruses or spike proteins from the cell surface. The cells were then washed, fixed, permeabilized, and treated with mouse monoclonal (1A9) antibody specific to SCV2 spike glycoprotein amino acid sequence 1000–1200 (Abcam, Cambridge, UK, cat. no. 273433). After incubation of 1 h at room temperature, the cells were then treated for 1 h at room temperature with either AF 488- or AF 633-labeled goat anti-mouse IgG (both Invitrogen, Carlsbad, CA, USA, cat. no. A-11001 and A-21052, respectively). For colocalization analyses of SCV2 or spike and SDCs, some cells were also incubated with APC-labeled SDC antibodies as described previously. The samples were then rinsed three times with PBS containing 1% BSA and 0.1% Triton X-100 and progressed toward flow cytometry. Cellular uptake and colocalization were then measured with flow cytometry using an Amnis FlowSight imaging flow cytometer. A minimum of 5000 events per sample were analyzed. Appropriate gating in a forward-scatter-against-side-scatter plot was utilized to exclude cellular debris and aggregates. Fluorescence analysis was conducted using the Amnis IDEAS analysis software. The Bright Detail Similarity (BDS) feature was used to measure colocalization between two signals. A BDS score of 2 or greater represents high overlap [30].

### 4.5. Cell Viability Measurements

The effect of the applied treatments on cell viability was assessed with the EZ4U cell proliferation assay (Biomedica Gmbh, Vienna, Austria, cat. no. BI-5000), according to the manufacturer’s instructions. Absorbance was measured with a BioTek Cytation 3 multimode microplate reader (Agilent Technologies, Santa Clara, CA, USA).

### 4.6. Quantification and Statistical Analysis

Results are expressed as means + standard error of the mean (SEM). Differences between experimental groups were evaluated using one-way analysis of variance (ANOVA) using the IBM^®^ SPSS^®^ software (version 27.0.1.0, IBM, Armonk, New York, NY, USA). Values of *p* < 0.05 were accepted as significant [19,21,22].

## Data Availability

Data are contained within the article or Supplementary Materials.

## Supplementary Materials

The following supporting information can be downloaded at: https://www.mdpi.com/article/10.3390/ijms25147534/s1.
