# Supplementary material for: Mutual Inhibition of Antithrombin III and SARS-CoV-2 Cellular Attachment to Syndecans: Implications for COVID-19 Treatment and Vaccination
Source: Int J Mol Sci. 2024 Jul 9;25(14):7534. doi: 10.3390/ijms25147534 (PMC11277105; doi:10.3390/ijms25147534)
Supplement: Supplementary file 1 [file ijms-25-07534-s001.zip › ijms-3087176-supplementary.pdf]

Supplementary Materials: Mutual Inhibition of Antithrombin III and SARS-CoV-2 Cellular Attachment to Syndecans: Implications for COVID-19 Treatment and Vaccination

Anett Hudák, Dávid Pusztai, Annamária Letoha, Tamás Letoha

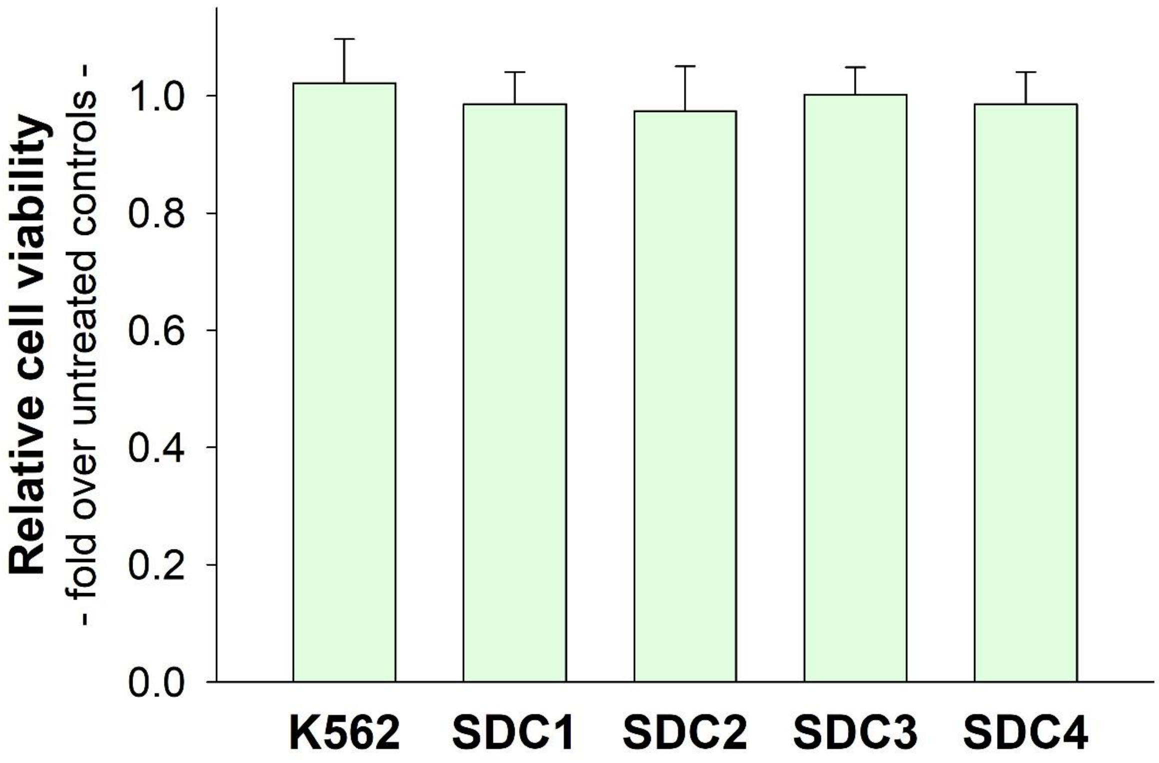

Figure S1. ATIII does not affect the cellular viability of K562 cells and SDC transfectants. WT K562 cells and stable SDC transfectants were incubated with or without 5 U/mL of recombinant ATIII for 30 min at 37°C. Cellular viability was then measured with the EZ4U assay, and detected measures were normalized to untreated cells as controls. The bars represent the mean + SEM of five independent experiments. Statistical significance vs. controls was assessed with ANOVA. Compared to controls, no statistically significant differences were detected in the viability of ATIII-treated cells.

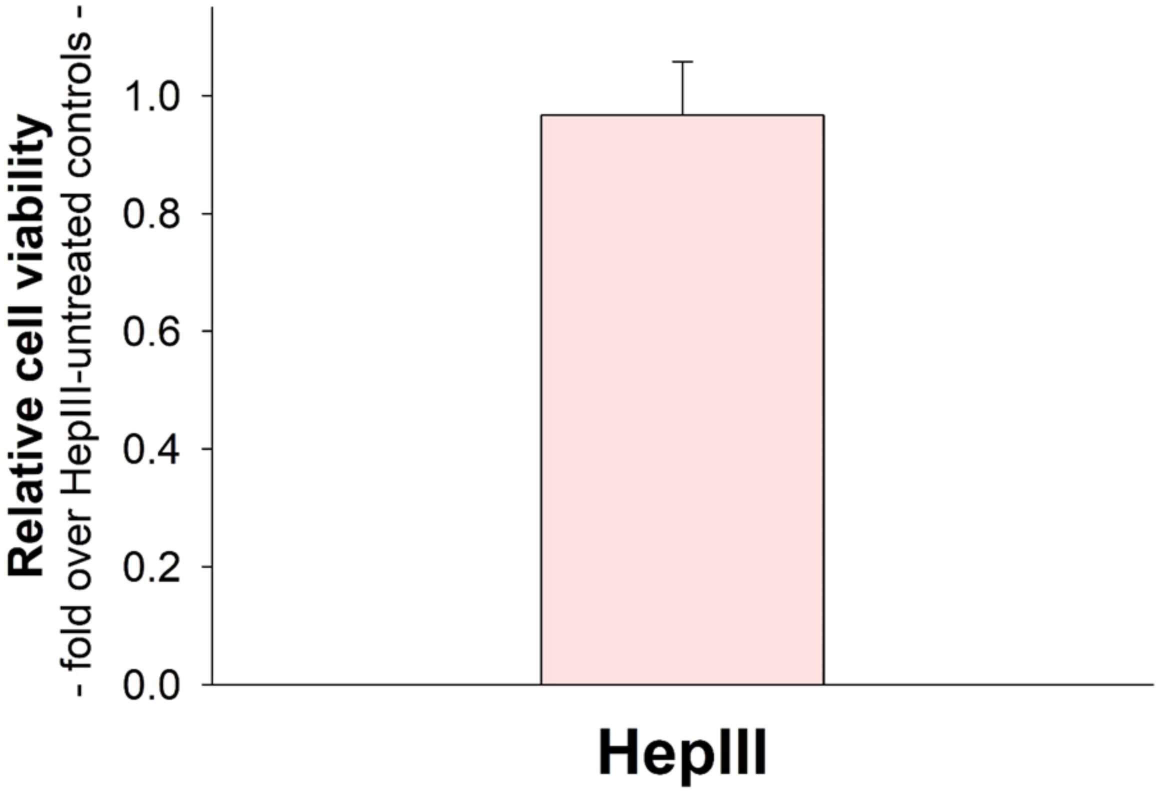

Figure S2. HepIII does not affect SDC4 transfectants' cellular viability. SDC4 transfectants were incubated with or without HepIII for 4 h at 37°C. Cellular viability was then measured with the EZ4U assay, and detected measures were normalized to HepIII-untreated cells as controls. The bars represent the mean + SEM of three independent experiments. Statistical significance vs. controls was assessed with ANOVA. Compared to controls, no statistically significant differences were detected in the viability of HepIII-treated cells.

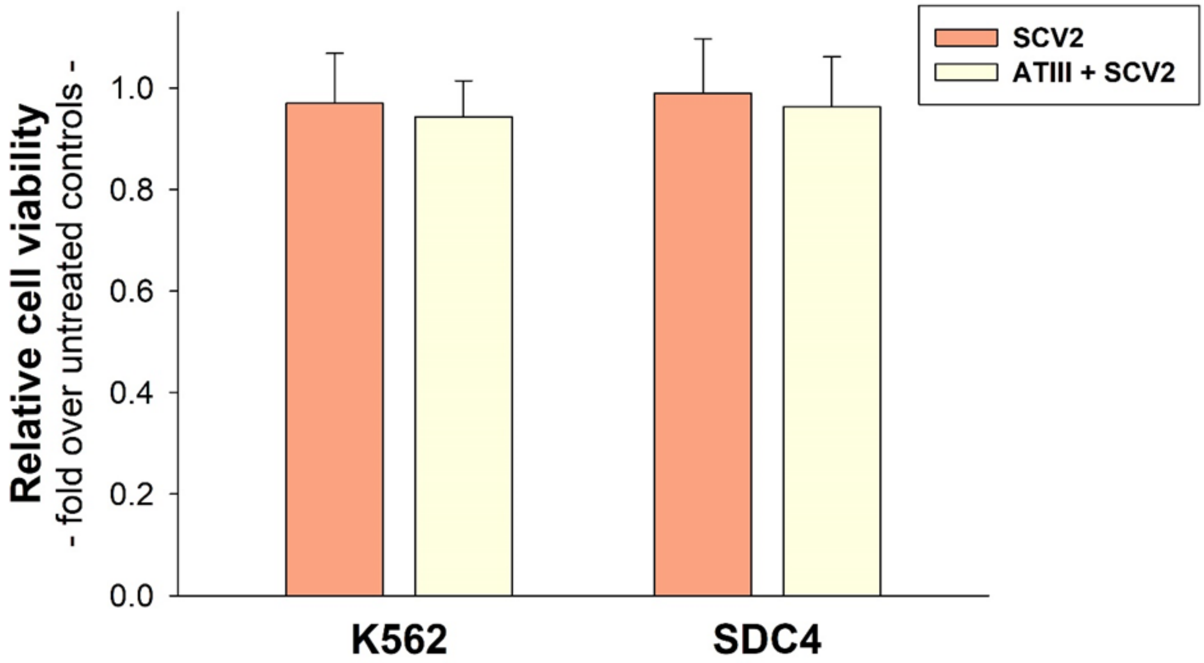

Figure S3. SCV2 does not affect the cellular viability of K562 cells and SDC4 transfectants at 1 MOI. WT K562 cells and stable SDC4 transfectants were exposed to 1 MOI of the heat-inactivated WT SCV2 with or without 5 U/mL of ATIII for 4 h at 37°C. Cellular viability was then measured with the EZ4U assay, and detected measures were normalized to untreated cells as controls. The bars represent the mean + SEM of three independent experiments. Statistical significance vs. controls was assessed with ANOVA. Compared to controls, no statistically significant differences were detected in the viability of SCV2-treated cells.

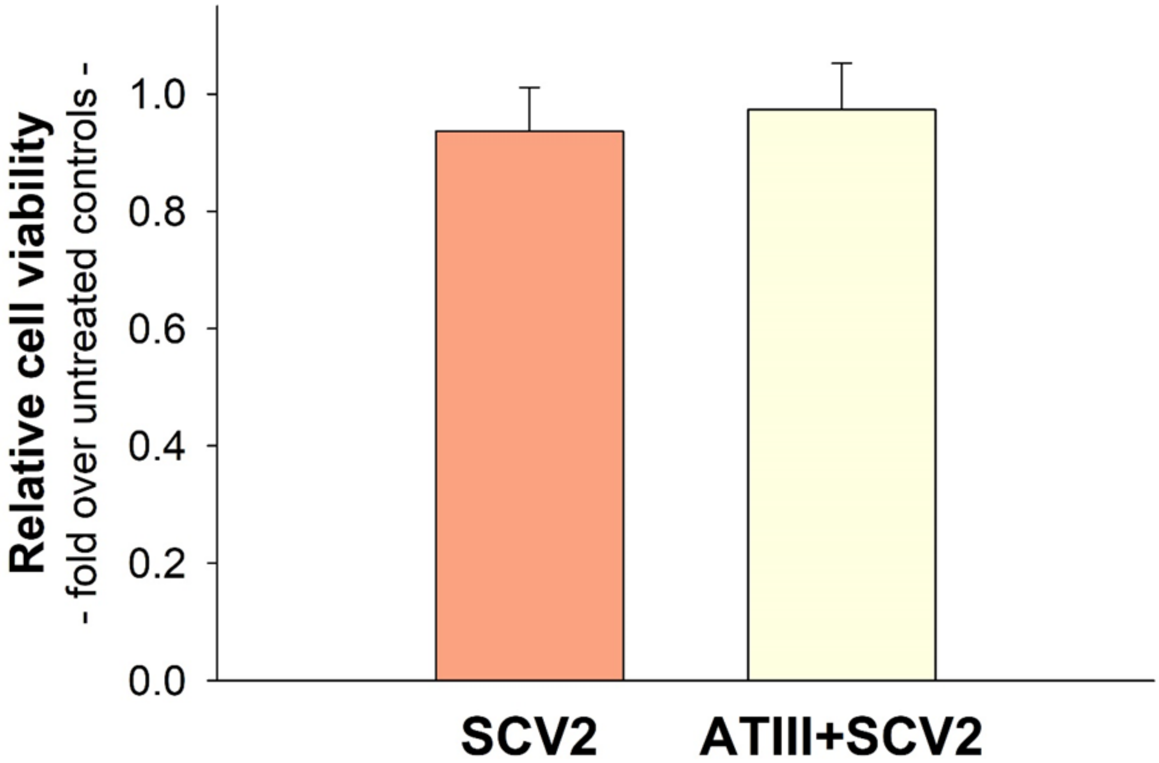

Figure S4. SCV2 does not affect the cellular viability of Calu-3 cells at 1 MOI. WT Calu-3 cells were exposed to 1 MOI of the heat-inactivated WT SCV2 with or without 5 U/mL of ATIII for 4 h at 37°C. Cellular viability was then measured with the EZ4U assay, and detected measures were normalized to untreated cells as controls. The bars represent the mean + SEM of three independent experiments. Statistical significance vs. controls was assessed with ANOVA. Compared to controls, no statistically significant differences were detected in the viability of SCV2-treated cells.

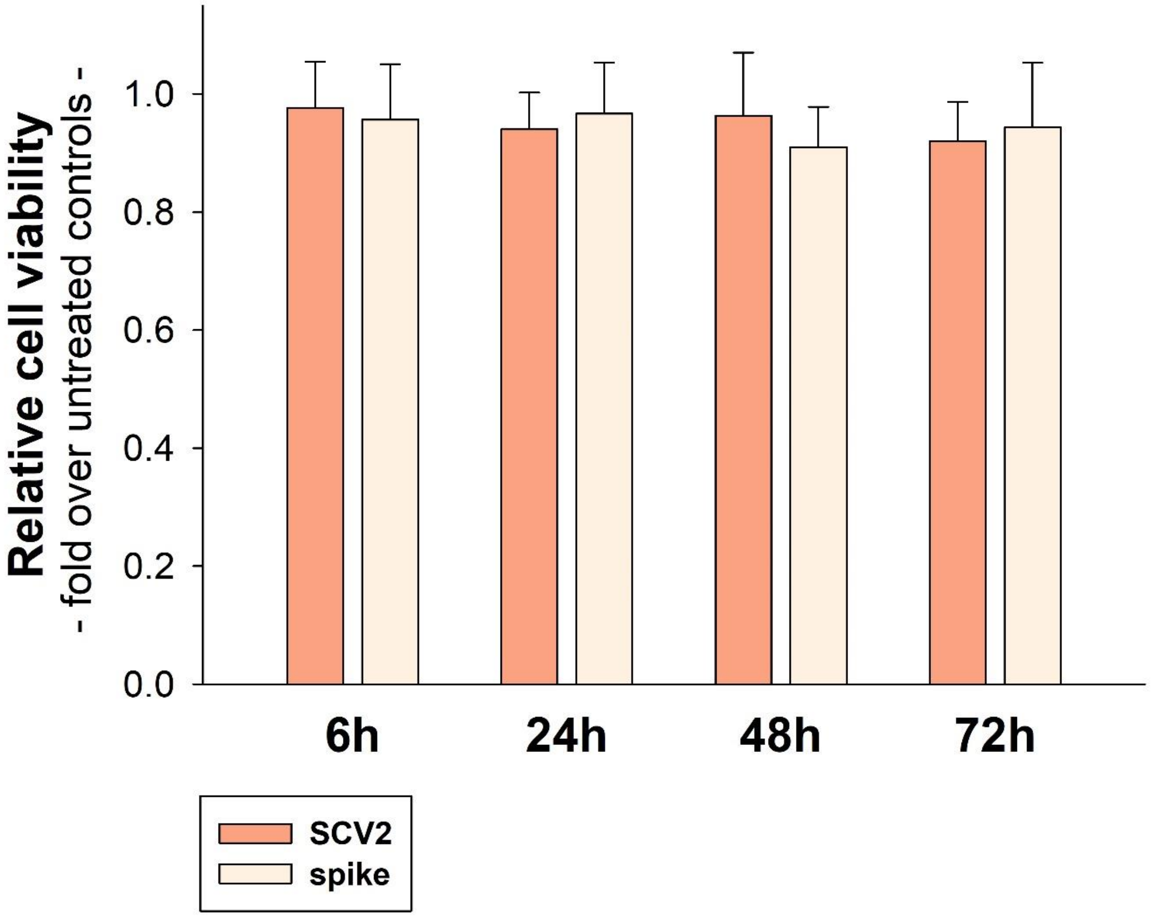

Figure S5. SCV2 or the spike protein does not affect the cellular viability of HMEC-1 cells. HMEC-1 cells were incubated with or without heat-inactivated SCV2 (1 MOI) or recombinant spike (5 µM) for 6, 24, 48 or 72 h at 37 °C. Cellular viability was then measured with EZ4U assay and detected measures were then normalized to untreated cells as controls. The bars represent the mean + SEM of three independent experiments. Statistical significance vs. controls was assessed with ANOVA. Compared to controls, no statistically significant differences were detected in the viability of SCV2-treated cells.
